# Innovative Design of a 3D Printed Esophageal Stent Inspired by Nature: Mitigating Migration Challenges in Palliative Esophageal Cancer Therapy

**DOI:** 10.3390/biomimetics9060359

**Published:** 2024-06-14

**Authors:** Thomas Profitiliotis, Savvas Koltsakidis, Konstantinos Tsongas, Dimitrios Tzetzis

**Affiliations:** 1 Digital Manufacturing and Materials Characterization Laboratory, School of Science and Technology, International Hellenic University, 57001 Thermi, Greece; 2 Department of Industrial Engineering and Management, School of Engineering, International Hellenic University, 57400 Thessaloniki, Greece; ktsongas@ihu.gr

**Keywords:** esophageal cancer, esophageal stenting, stent migration, stent complications, bioinspired stents, FEA

## Abstract

Esophageal cancer is a complex and challenging tumor to treat, with esophageal stenting being used as a palliative measure to improve the quality of life of patients. Self-expandable metal stents (SEMS), self-expandable plastic stents (SEPS), and biodegradable stents are the most commonly used types of stents. However, complications can arise, such as migration, bleeding, and perforation. To address issues of migration, this study developed a novel 3D printed bioinspired esophageal stent utilizing a highly flexible and ductile TPU material. The stent was designed to be self-expanding and tubular with flared ends to provide secure anchorage at both the proximal and distal ends of the structure. Suction cups were strategically placed around the shaft of the stent to prevent migration. The stent was evaluated through compression–recovery, self-expansion, and anti-migration tests to evaluate its recovery properties, self-expansion ability, and anchoring ability, respectively. The results indicated that the novel stent was able to recover its shape, expand, keep the esophagus open, and resist migration, demonstrating its potential for further research and clinical applications. Finite element analysis (FEA) was leveraged to analyze the stent’s mechanical behavior, providing insights into its structural integrity, self-expansion capability, and resistance against migration. These results, supported by FEA, highlight the potential of this innovative stent for further research and its eventual application in preclinical settings.

## 1. Introduction

Esophageal cancer is considered to be one of the most difficult and complicated tumors to treat and is the sixth most common cause of cancer-related deaths globally [1]. Around 15–34% of esophageal cancer patients survive the disease for 5 years [2]. In such cases, esophageal stenting can be used as a palliative measure to provide relief from symptoms and improve quality of life [3]. The stent is placed in the esophagus to create an artificial opening, allowing food and liquids to pass more easily and reducing the symptoms of difficulty in swallowing (dysphagia).

Esophageal stents have a relatively long history of use, with the first reported use of a stent for esophageal stricture dating back to the 1970s [4]. Since then, the design and materials of esophageal stents have evolved, and their use has become increasingly common for a variety of clinical applications, including the management of malignant and benign esophageal strictures and esophageal perforation [5,6,7]. Self-expandable metal stents (SEMS) were first introduced as a treatment option for malignant esophageal strictures and were designed to address some of the limitations of the earlier, non-expandable metal stents [8]. SEMS, predominantly composed of nitinol or stainless steel, consist of a metal mesh or wire structure that is compressed for insertion and then expands to its final shape once it is in place [9,10]. Despite the increased usage of SEMS in clinical settings in recent decades because of their easy placement, complications have arisen during either the early or late stages of the procedure. The difficulties arising from this include the overgrowth of tumor tissue, bleeding, the reappearance of constrictions, the perforation of tissues, gastroesophageal reflux, and the displacement of the stent into the stomach [11]. With the introduction of plastic and silicone, self-expandable plastic stents (SEPS) arose. Unlike SEMS, SEPS are made of plastic materials such as polyethylene and flexible silicones, which are biocompatible materials [12]. SEPS are thought to have several advantages over SEMS, including lower cost, a reduced risk of migration or dislodgement, and the ability to be removed if necessary [13]. However, SEPS may have a shorter lifespan and may not be as durable as SEMS in some cases. With the success rates for benign strictures ranging widely from 17% to 95%, SEPS are now widely used in the treatment of benign conditions such as refractory strictures, perforations, fistulas, and anastomotic leaks [14,15]. Over the last decade, there has been significant interest in biodegradable stents. Unlike traditional stents, which are made from metal or plastic and are designed to remain in the body permanently, biodegradable stents are made from biodegradable poly-L-lactic monofilaments or polydioxanone (PDS), which gradually dissolve or degrade over time [16,17,18,19,20,21]. The main advantage of biodegradable esophageal stents is that they allow the natural healing and restoration of the esophagus, reducing the risk of long-term complications that can occur with permanent stents. Despite their advantages, biodegradable stents also have some drawbacks. Similar to commercially available SEMS and SEPS, biodegradable stents tend to have a high incidence of stent migration during the early stages [22].

Thermoplastic polyurethane (TPU) presents an appealing choice for esophageal stents due to its flexibility and biocompatibility [23]. Advancements in TPU technology have led to the development of biodegradable grades, which gradually degrade over time within the body. These biodegradable TPUs are designed to break down into non-toxic byproducts, eliminating the need for surgical removal and reducing the risk of the long-term complications associated with permanent implants [24].

The specific fabrication processes used to manufacture esophageal stents vary, depending on the type of stent being made, the materials used, and the manufacturer’s procedures. Some common fabrication processes used in manufacturing esophageal stents include laser cutting, injection molding, and welding [25,26,27]. Fused filament fabrication (FFF) is an additive manufacturing technique that involves the successive addition of layers of material to produce a tangible object until it is finished. This process has the potential to revolutionize the manufacturing of esophageal stents by providing greater design freedom and faster production times compared with traditional manufacturing methods [28]. Three-dimensional printing can be used to produce custom-sized and shaped stents that fit the unique anatomy of each patient [29]. This can be particularly beneficial for patients with complex or challenging anatomies, such as those with a narrow or irregular esophagus.

Biomimicry in design is a field that draws inspiration from nature to create sustainable and innovative solutions to various challenges [30]. The underlying concept of this method is that the natural world has created proficient and productive systems through millions of years of evolution, and that these systems can offer useful perspectives on addressing issues in engineering, architecture, and other domains [31,32,33]. In biomimicry, nature is viewed as a model, mentor, and measure, and designers attempt to replicate the underlying principles and functions of natural systems in their work [34]. *Taenia solium* is a parasitic tapeworm that can infect humans. It is commonly known as the pork tapeworm because its larvae can be found in the flesh of infected pigs [35]. The scolex of *Taenia solium* is a distinct feature of the parasite that is composed of several hooks and suckers, which allow it to attach to the wall of the small intestine [36]. These hooks and suckers help the parasite to resist the peristaltic movement of the intestine and maintain its position within the host.

This study developed a new bioinspired tubular esophageal polymeric stent to resolve the problem of migration often seen in current stents. The stent’s external surface imitates the scolex of a tapeworm to keep it in place in the esophagus. By utilizing bothridia, which are the cup-shaped protruding chambers found in *Taenia solium*, the stent can conformably attach to diverse surfaces through its force of self-expansion [37]. The esophageal stent consists of a thermoplastic polyurethane (TPU) material. It is a versatile material that combines the properties of both rubber and plastic. TPU has high elongation, excellent tear strength, and provides the necessary flexibility [38] for ease of deployment and self-expansion force. It is often used in biomedical applications that require both flexibility and durability [39]. As a result of these characteristics, TPU has been utilized to develop and produce various tubular esophageal stents [40]. However, the application of bothridia in the development of esophageal stents has yet to be explored.

The objective of this investigation was to develop a stent for the esophagus by utilizing FFF technology, TPU material, and bothridia. The stent needed to have the capability to be compressed into a stent delivery system, expand and open a cancer-induced blocked esophagus, and minimize the possibility of migrating into the stomach. The properties of self-expansion and anti-migration of the 3D printed stent were assessed. The results obtained through finite element analysis (FEA) were encouraging, as they suggested that the novel 3D printed stent could successfully regain its original shape, expand to ensure the openness of the esophagus, and demonstrate robust resistance against migration.

## 2. Materials and Methods

### 2.1. Design

#### 2.1.1. The Patient-Specific Esophageal Stent

**Figure 1:**
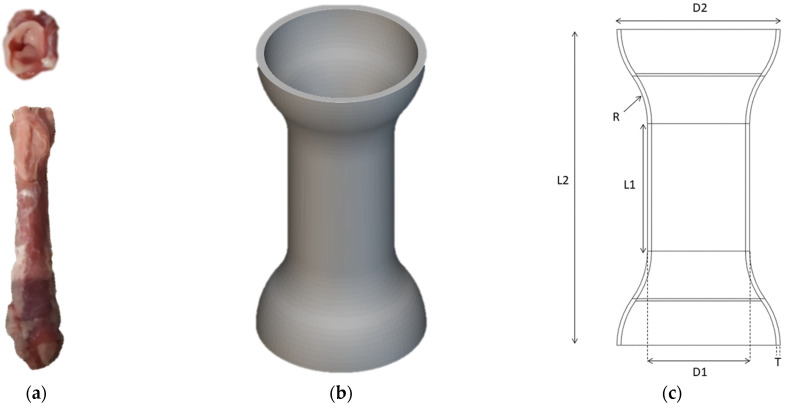

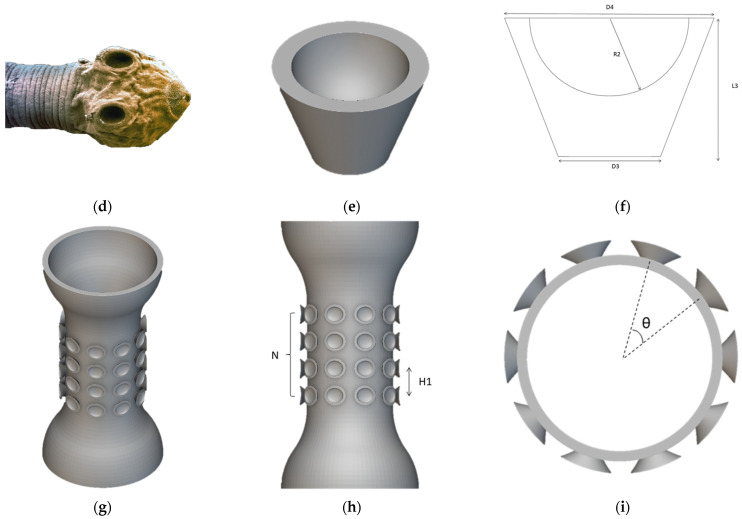
(**a**) Harvested porcine esophagus. (**b**) Parametric model of the stent. (**c**) The stent’s design parameters. (**d**) A tapeworm’s scolex bothridia. (**e**) Parametric model of a suction cup. (**f**) the suction cup’s design parameters. (**g**) Parametric model of the bioinspired stent. (**h**) The bioinspired stent’s design parameters (front view). (**i**) The bioinspired stent’s design parameters (section view).

A patient-specific esophageal stent was designed using SolidWorks 2022 (Dassault Systèmes SolidWorks Corp., Waltham, MA, USA), based on the dimensions of a porcine esophagus (Figure 1a). The stent was designed to be self-expanding and tubular with flared ends (Figure 1b) to provide secure anchorage at both the proximal and distal ends of the stricture. The design was optimized using computer-aided design techniques to ensure appropriate dimensions and geometries. The simple stent’s design variables are detailed in Table 1 and Figure 1c.

#### 2.1.2. Suction Cup

In order to create an efficient suction cup, the design was inspired from nature, namely the structure of the tapeworm’s bothridia (Figure 1d), which are known to have a highly efficient attachment mechanism. Using nTopology 5.6 (nTopology Inc., New York, NY, USA), a suction cup (Figure 1e) was designed that mimicked the structure of the bothridium. By replicating the features of the bothridium, the aim was to create a suction cup that could achieve a similar level of attachment. The dimensions of the suction cup (Figure 1f) were carefully chosen to optimize its function. 

#### 2.1.3. The New Bioinspired Esophageal Stent

**Figure 2:**
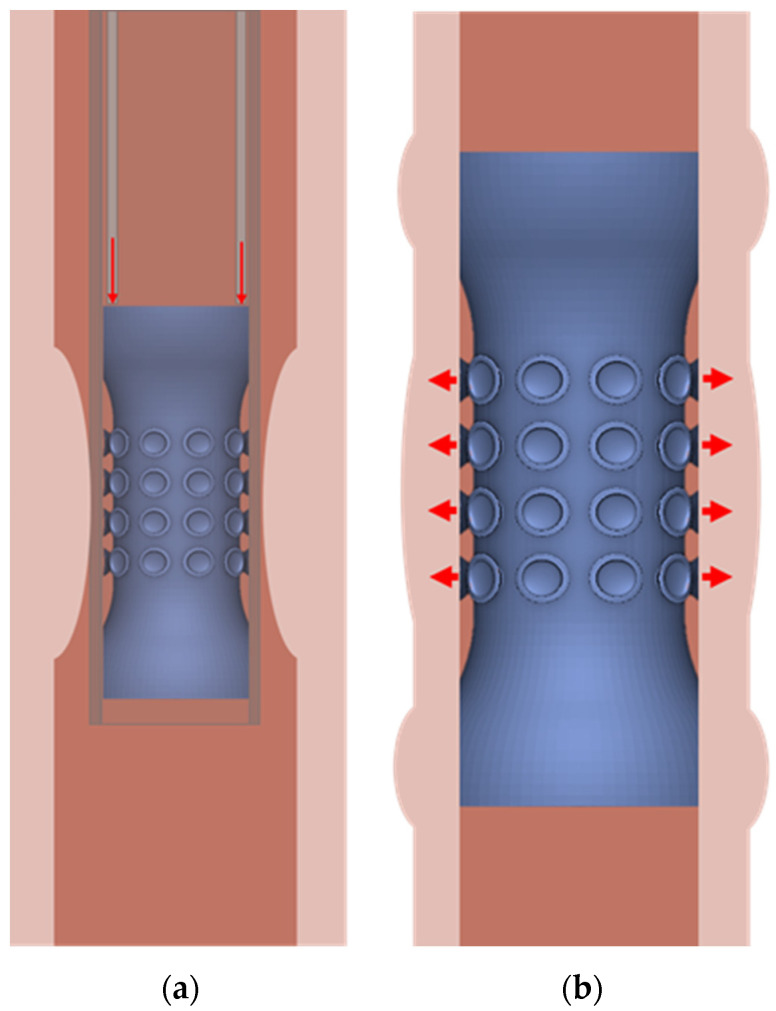
(**a**) Deployment of the bioinspired stent. (**b**) The bioinspired stent’s mechanism of self-expansion and adhesion.

The new bioinspired esophageal stent was created using nTopology (Figure 1g). The external surface of the stent was designed to mimic the tapeworm’s scolex for better anchoring to the esophageal tissue. Suction cups were strategically placed around the shaft of the stent to prevent migration. The exact dimensions of the suction cups and their positioning relative to the stent are detailed in Figure 1h,i, and Table 2 and Table 3. Figure 2 presents a graphical representation of the stent’s deployment in (a) and illustrates the self-expansion and adhesion mechanism of the bioinspired stent in (b).

### 2.2. Fabrication

**Figure 3:**
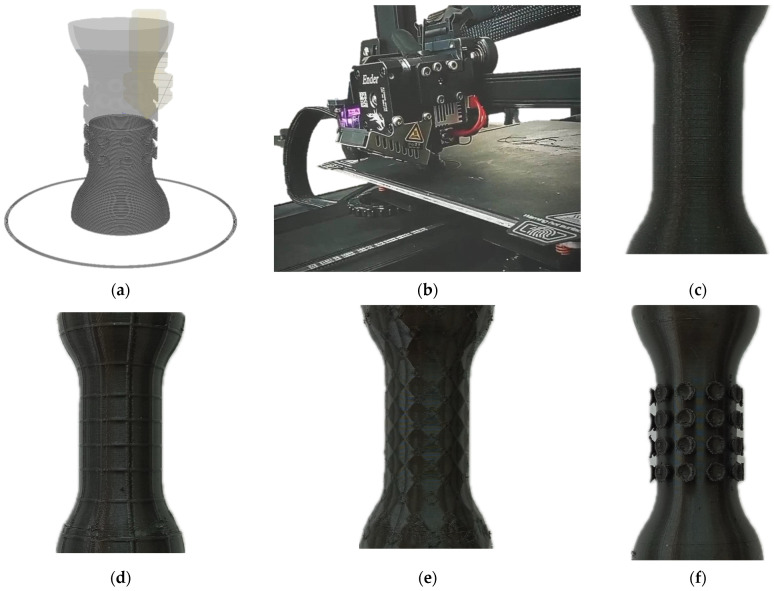
(**a**) File preparation stage. (**b**) Fabrication process. (**c**) Simple 3D printed stent. (**d**) The 3D printed SC stent. (**e**) The 3D printed FCC stent. (**f**) Bioinspired 3D printed stent.

A highly flexible, ductile, and not rapidly yet still inherently degradable TPU filament (1.75 mm diameter), made from varioShore TPU (ColorFabb BV, Limmburg, The Netherlands), was obtained. This specialized TPU allows users to vary the density from 0.7 to 0.9 g/cm^3^ by adjusting the printing temperature across 200–250 °C and the flow rate across 60–70%. At higher temperatures (200–250 °C) the material actively foams, expanding 1.4–1.6× its original volume to produce very soft prints with Shore A hardness down to ~55 A. Printing at lower temperatures (190–200 °C) suppresses foaming, resulting in an unfoamed solid with higher density and Shore ~92 A hardness. TPU-based materials show low cytotoxicity and good compatibility with living cells, making them suitable for cell imaging and tissue engineering applications [41,42]. During the file preparation stage, the design files for the new bioinspired esophageal stent were prepared using Ultimaker Cura 5.5.0 software (Ultimaker BV, Utrecht, The Netherlands) (Figure 3a). The software was used to generate the tools’ path and the slicing information required for 3D printing. The stent design was optimized for printing using a fused filament fabrication (FFF) 3D printer. The necessary parameters (Table 4), such as the print speed, layer height, and infill density, were adjusted to ensure that the stent was accurately and efficiently printed. In the fabrication stage, the new bioinspired esophageal stent was 3D printed using the fused filament fabrication (FFF) technique (Figure 3b). The FFF technique involved the extrusion of TPU layer by layer to build the stent. The printer used for this process was the Creality Ender-3 S1, which offers high precision and accuracy in printing. Once the printing was complete, the stents were removed from the printer bed and cleaned of any excess material or support structures. The stents were then sterilized before being used for evaluation. As well as the bioinspired stent, some other designs were 3D printed, such as a simple stent without any surface features. The simple cubic (SC) stent featured a basic lattice structure, while the face-centered cubic (FCC) stent showcased a more complex lattice arrangement for enhanced mechanical properties and surface characteristics (Figure 2c–f).

### 2.3. Evaluation

#### 2.3.1. Compression–Recovery Test

**Figure 4:**
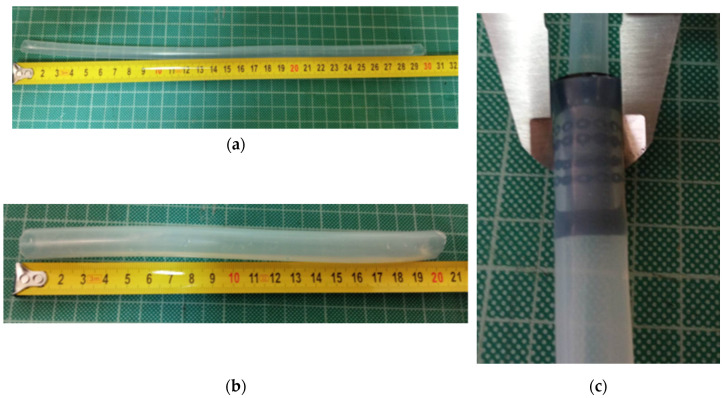
(**a**) Silicon tube, Φ6 × 9. (**b**) Silicon tube, Φ10 × 14. (**c**) Compression–recovery test.

In this test, the stent was compressed in the stent delivery system, which consists of two silicon tubes with different diameters. The initial silicon tube, measuring 6 mm in diameter (Figure 4a), was used to load and unload the stent into the larger silicon tube, which had a diameter of 10 mm (Figure 4b). The compression–recovery test was conducted using a sample size of 5 to evaluate each stent’s configuration and recovery properties after being compressed in the delivery system (Figure 4c). This test is an essential method in the development of esophageal stents to ensure that the stent has enough flexibility and ability to recover to keep the esophagus open and prevent collapse. After being loaded for a few minutes, the stent was pushed out from the delivery system. The compression–recovery test method is a crucial parameter to ensure the stent’s effectiveness in clinical applications. 

#### 2.3.2. Self-Expansion Test

**Figure 5:**
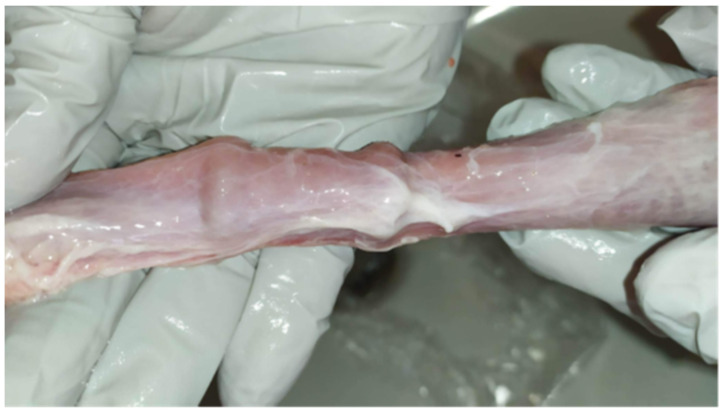
Self-expansion test.

In the self-expansion test, the stent’s ability to expand and keep the esophagus open was evaluated. In total, 5 samples of all of the stent’s configurations were deployed inside a porcine esophagus strip (Figure 5), which simulated the in vivo environment of the human esophagus, using the stent delivery system. The size of the opened esophagus was then measured to evaluate the self-expansion property of the stent. The porcine esophagus used in the test was obtained from a local slaughterhouse in Greece (Sfageia Edessis S.A., Edessa, Greece). This test is an important step in the development of esophageal stents, as it helps to ensure that the stent is able to expand and maintain the patency of the esophagus in a realistic environment.

#### 2.3.3. Anti-Migration Force Test and Finite Element Analysis

**Figure 6:**
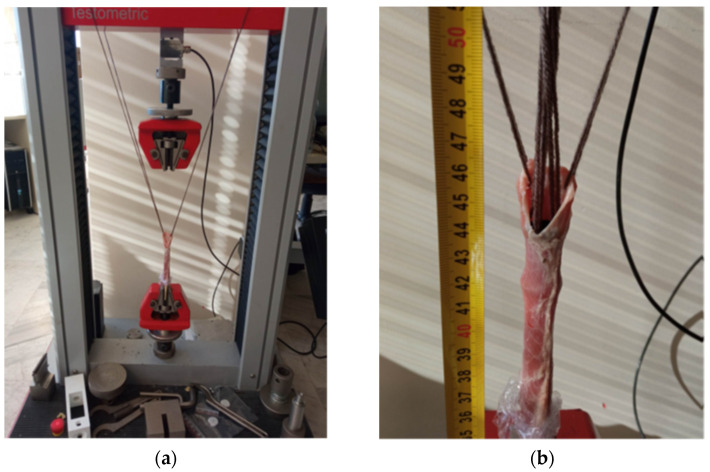
(**a**) Experimental setup of the anti-migration test; (**b**) anti-migration force test.

In the anti-migration test, the stent’s anchoring ability and resistance to migration were evaluated. To perform this test, the stent was deployed inside the esophageal strip using the stent delivery system, and one end of the strip was clamped into the tensile tester’s gripper. (Figure 6a) The other end was held open with the help of a thread, and threads were used to subject the stent to a tensile load. (Figure 6b) The force required to dislodge the stent was measured, which was an indicator of the stent’s anchoring ability. This test is important to reduce the risk of the stent’s migration and to ensure its stability in the esophagus. The tests were examined on a Testometric M500-50AT system (Testometric Ltd., Rochdale, UK) with a constant deformation rate of 5 mm/min for all configurations. The anti-migration tests were performed on a total of 5 samples per stent configuration to ensure the reliability of the results.

The anti-migration properties of various stent designs were evaluated through both experimental and finite element analysis (FEA). ANSYS™ 2023 R2 software (ANSYS, Inc., Canonsburg, PA, USA) was utilized to study the mechanical behavior of the conventional, lattice, and biomimetic stents. FE models were integrated to enhance the accuracy of the force calculations by effectively replicating the anti-migration assessment. An explicit dynamic analysis was performed to accurately simulate the mechanical response of the stents, capturing the extensive deformations and hyperelastic material characteristics of the esophagus. The multilayered skin model of the porcine esophagus comprised the mucosa–submucosa and muscle layers, each with specific mechanical properties obtained from tensile tests. The stents were modeled using a five-parameter Mooney–Rivlin material model, with the parameters fine-tuned to align the simulation’s results with experimental data. The simulations included material erosion to account for potential slippage and failure. The comprehensive FE parameters, such as the material constants and boundary conditions, are fully explained and presented in the Results section (Section 3.3, Anti-Migration Test Results assisted by FEA). The FEA results closely matched the experimental forces, validating the model and confirming the superior performance of the bioinspired stent in anti-migration tests. 

#### 2.3.4. Tensile Tests

The TPU material underwent rigorous tensile testing, using dogbone-shaped specimens prepared in adherence to ISO 527-1:2019 [43]. In total, five specimens of all configurations were subjected to controlled rates of deformation using the Testometric M500-50AT system. The resulting stress–strain curves and mechanical parameters, including tensile strength and elongation at breaking, were systematically recorded. This thorough analysis characterized the TPU material’s behavior under tensile loading, providing valuable insights into its structural integrity and performance concerning esophageal stent applications. 

**Figure 7:**
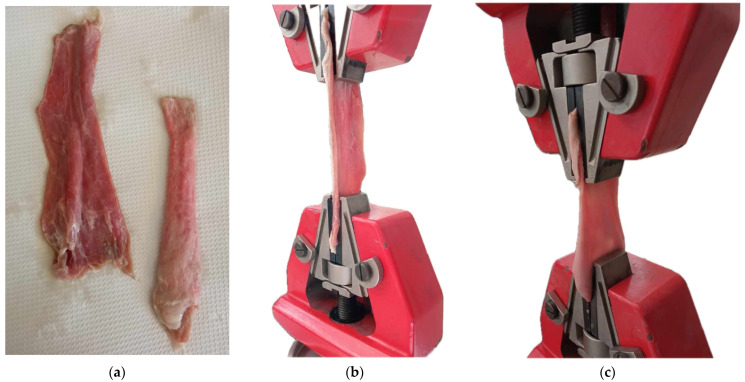
(**a**) Muscle and mucosa–submucosa layers. (**b**) Tensile test of the muscle layer. (**c**) Tensile test of the mucosa–submucosa layer.

The mechanical properties of the porcine esophagus, serving as a surrogate for human tissue, were assessed through tensile testing of two distinct flattened layers: the mucosa–submucosa and muscle layers (Figure 7a). Specimens, meticulously prepared from fresh porcine esophagi, underwent tensile tests using the Testometric M500-50AT system (Figure 7b,c). The acquired stress–strain curves and mechanical data offered a detailed understanding of the esophageal tissue’s response to tensile forces. This investigation, which was crucial for ensuring that the 3D printed esophageal stent aligned with the biomechanical characteristics of native tissue, minimized the risk of adverse reactions and optimized the implant’s long-term functionality.

## 3. Results

### 3.1. Compression–Recovery Test Results

**Figure 8:**
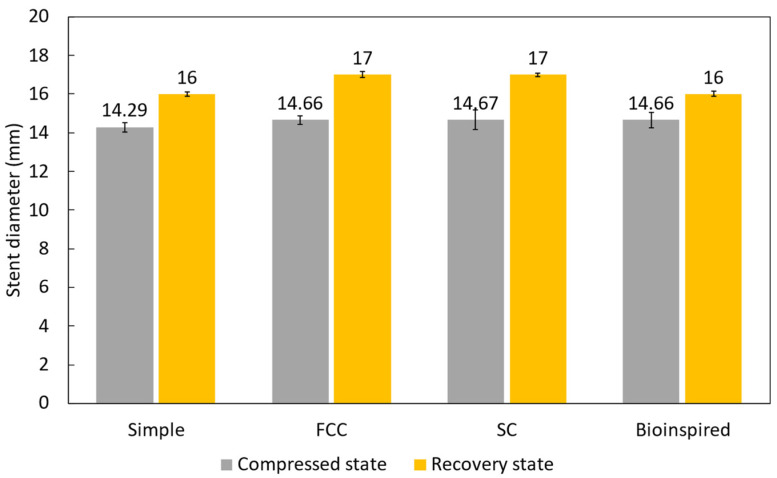
Evaluation of compression–recovery properties.

In the compression–recovery test, the stent’s ability to withstand external pressure and then recover its original shape was evaluated. The results obtained (Figure 8) showed that the stent had a compressed state of 14.29 mm and a recovery state of 16 mm. The FCC stent had a compressed state of 14.66 mm and a recovery state of 17 mm, while the SC stent had a compressed state of 14.67 mm and a recovery state of 17 mm. The new bioinspired stent had a compressed state of 14.66 mm and a recovery state of 16 mm. These results indicated that all stents were able to withstand compression to a similar extent and recover their original shape, suggesting good mechanical stability and indicating that no permanent deformation or damage occurred during compression. 

### 3.2. Self-Expansion Test Results

**Figure 9:**
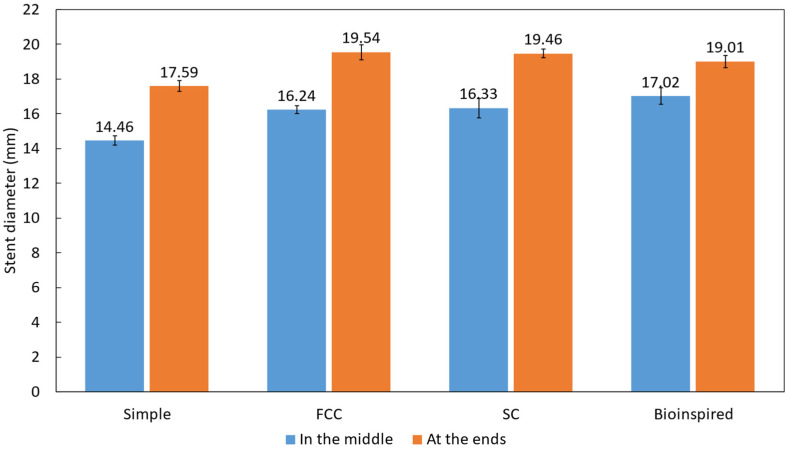
Evaluation of the self-expansion properties.

The results (Figure 9) showed that the simple stent had the smallest expanded diameter in the middle of the stent (14.46 mm) and at the ends of the stent (17.59 mm) among the four stents tested. This suggests that the self-expansion property of the simple stent may be weaker than the others, which could limit its ability to keep the esophagus open. The FCC stent had a larger expanded diameter in the middle of the stent (16.24 mm) and at the ends of the stent (19.54 mm) than the simple stent, indicating better self-expansion ability. The SC stent had a similar expanded diameter in the middle of the stent (16.33 mm) to the FCC stent but a slightly smaller diameter at the ends of the stent (19.46 mm). The new bioinspired stent had the largest expanded diameter in the middle of the stent (17.02 mm) among the four stents tested, indicating strong self-expansion ability. However, its expanded diameter at the ends of the stent (19.01 mm) was smaller than that of the SC and FCC stents.

### 3.3. Anti-Migration Test Results Assisted by FEA

**Figure 10:**
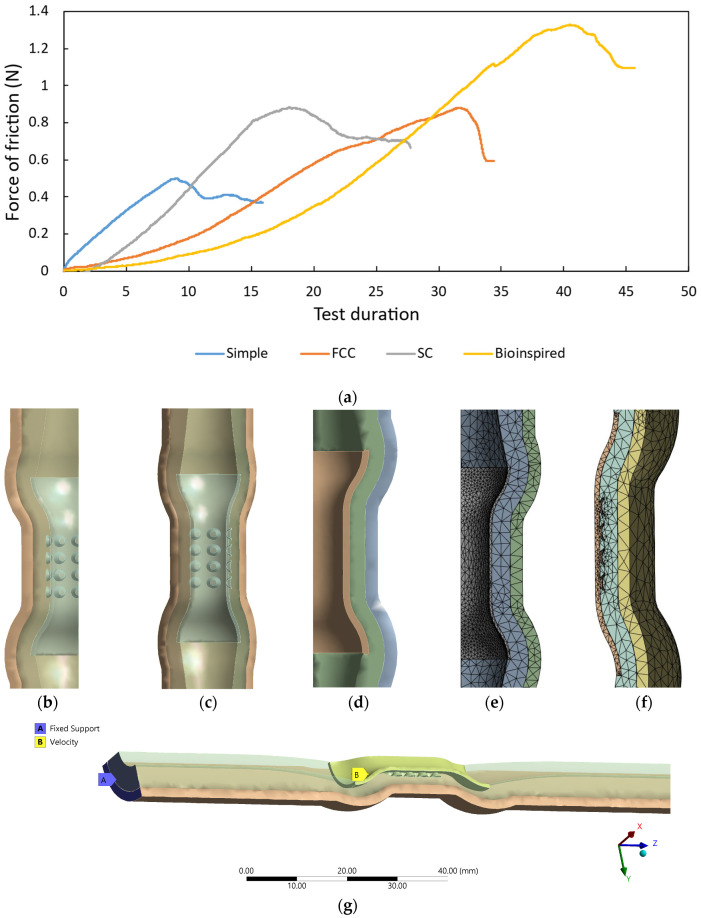
Evaluation of anti-migration properties: (**a**) force friction curves, (**b**–**d**) views of the bioinspired stent, (**e**,**f**) the mesh model of the bioinspired stent, and (**g**) typical boundary conditions.

In the anti-migration test, the anchoring ability of each stent was evaluated by measuring the anti-friction force required to prevent slippage. The experimental test results, as depicted in Figure 10a, showed that the simple stent required 0.5 N of force to prevent slippage. The FCC and SC stents demonstrated improved anchoring ability, requiring 0.88 N and 0.87 N of force, respectively. The novel bioinspired stent showed the highest anchoring ability, requiring 1.32 N of force to prevent slippage. Notably, the 1.32 N value for the bioinspired stent approached the optimal ~1.5 N frictional force suggested by Maohua Lin et al. for effective anti-migration in esophageal stenting applications [44]. These results suggest that the novel stent may be the most effective at reducing the risk of migration.

FEA models were introduced to enhance the accuracy of the force calculations, as they can precisely simulate complex mechanical systems. In tests such as the anti-migration assessment, it is crucial to measure the exact force required to dislodge the stent for evaluating its anchoring ability. FEA contributes to a comprehensive understanding of these mechanical properties, consequently securing the stent’s stability and mitigating the risk of migration. An explicit dynamic analysis was executed using ANSYS software to study the force necessary to counteract slippage. A multilayered skin model, comprising two distinct layers, namely the muscle and mucosa layers of the porcine esophagus, was deemed to be suitable. Further research [45,46,47] on a similar subject has demonstrated that using an FEA-assisted mechanical testing approach can effectively align with experimental data and derive a precise model to characterize the system’s performance.

A schematic representation of the FEA model is illustrated in Figure 10b–d, where the two distinct stent configurations are prominently illustrated, complete with the specified boundary conditions and the representation of the esophagus’ two layers. More specifically, the first layer represents the mucosa–submucosa and has been given a thickness of 5 mm; the second layer is equivalent to the muscle with 2 mm. Symmetric boundary conditions have been applied to efficiently model the entire structure while minimizing the computational demands. Specifically, one-fourth of the structure was meshed, and then symmetry was enforced around two planes, ensuring that the analysis accurately reflected the system’s behavior with reduced computational resources. A convergence study was conducted to verify that the response was independent of the mesh, revealing that stress convergence was achieved with nearly 100,000 elements for each validation model (Figure 10e,f).

The introduction of material failure and the development of a contact surface between the stent and the target were used to depict the potential slippage of the stent’s array of suction cups on the surface of the esophagus. This behavior was influenced by substantial deformations. An algorithm for material erosion was utilized to analyze the failure and separation of the stent’s material. This material erosion procedure removed distorted elements during the solution, particularly along the stent’s path, based on material failure and the potential for separation caused by fracturing of the esophagus’ surface.

The starting values for the mechanical properties of the mucosa–submucosa and muscle used in these simulations were obtained from the tensile test of the porcine esophagus. The material parameters were then fine-tuned to match the FEA force–depth data with the experimental results. Consequently, the process of identifying these parameters could be regarded as a minimization problem. The FEA model required material properties specific to the filament utilized in creating the 3D printed stents. The TPU’s tensile strength was established at 20 ± 5 MPa, and its elastic modulus was determined to be 71 MPa via compression and nanoindentation mechanical testing methods [45,46,47]. 

**Figure 11:**
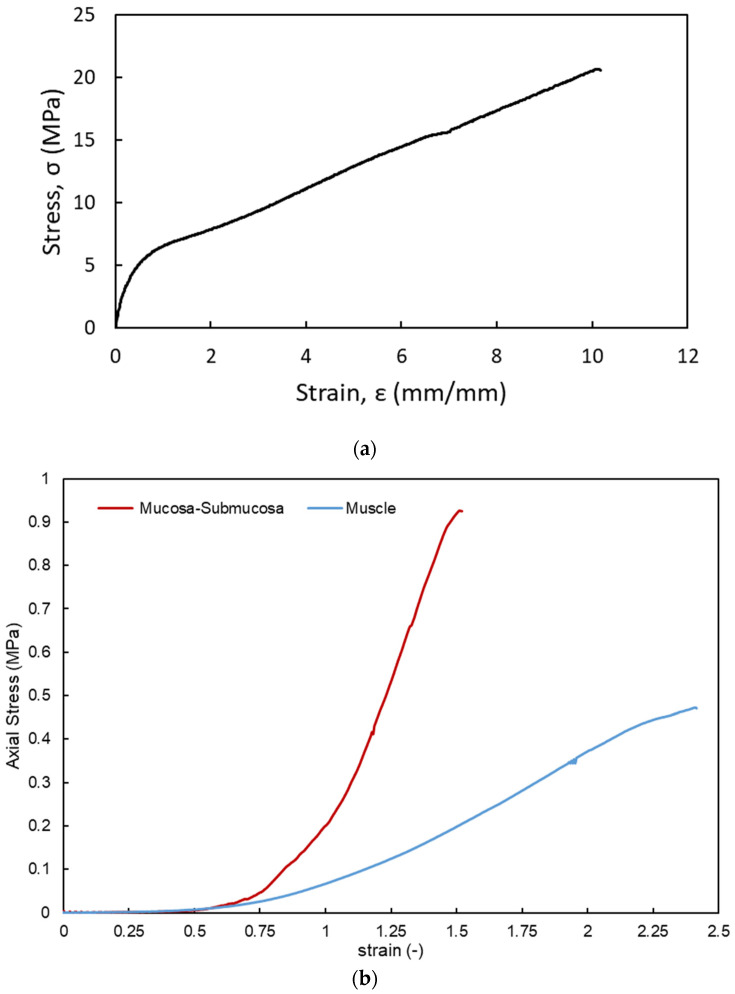
(**a**) Tensile test of the TPU specimen. (**b**) Tensile test of the porcine esophagus to determine the stress–strain properties of the mucosa–submucosa and muscle for the FE model.

This research used a five-parameter incompressible Mooney–Rivlin material model, designed to characterize the behavior of tissue-like materials at a local level. It was assumed that the local strain energy density within an incompressible solid could be described as a straightforward function of the invariant coefficients of local strain. These invariant coefficients were determined by fitting the simulation model to a conventional tensile test. The general form of the five-parameter Mooney–Rivlin model is given by the following equation

(1) $$W=C_{10}\left(\bar{I_{1}}-3\right)+C_{01}\left(\bar{I_{2}}-3\right)+C_{11}\left(\bar{I_{1}}-3\right)\left(\bar{I_{2}}-3\right)+C_{20}{\left(\bar{I_{1}}-3\right)}^{2}+C_{02}{\left(\bar{I_{2}}-3\right)}^{2}+\frac{1}{D}{\left(J-1\right)}^{2}$$

where W is the function of strain energy, $\bar{I_{1}}$ and $\bar{I_{2}}$ are the first and second strain invariants, $C_{ij}$ represents the material constants that have been regulated properly to match the non-linear sections of the stress–strain diagram, D is the material’s incompressibility parameter, and J represents the Jacobian determinant, which is a measure of the volume of change in a deformation. The constants $C_{ij}$ are material parameters that are determined by fitting Equation (1) to the experimental data from the tensile test. In order to obtain these values, the tensile strength of the stent’s TPU material, along with the muscle and mucosa–submucosa layers of the porcine esophagus, were determined by tensile testing, as shown in Figure 11. The parameters were obtained from the Mooney–Rivlin model and are listed in Table 5. These parameters were used for the computational simulation of the anti-migration behavior.

The results of the simulation closely matched the experimental force data, with maximum forces reaching 0.4 N, 0.78 N, 0.7 N, and 1.54 N for the simple, FCC, SC, and novel bioinspired stents, respectively. Since the material parameters in the FEA model aligned with the experimental data, the parameters of the hyperelastic material model for the esophagus were considered to be optimal.

**Figure 12:**
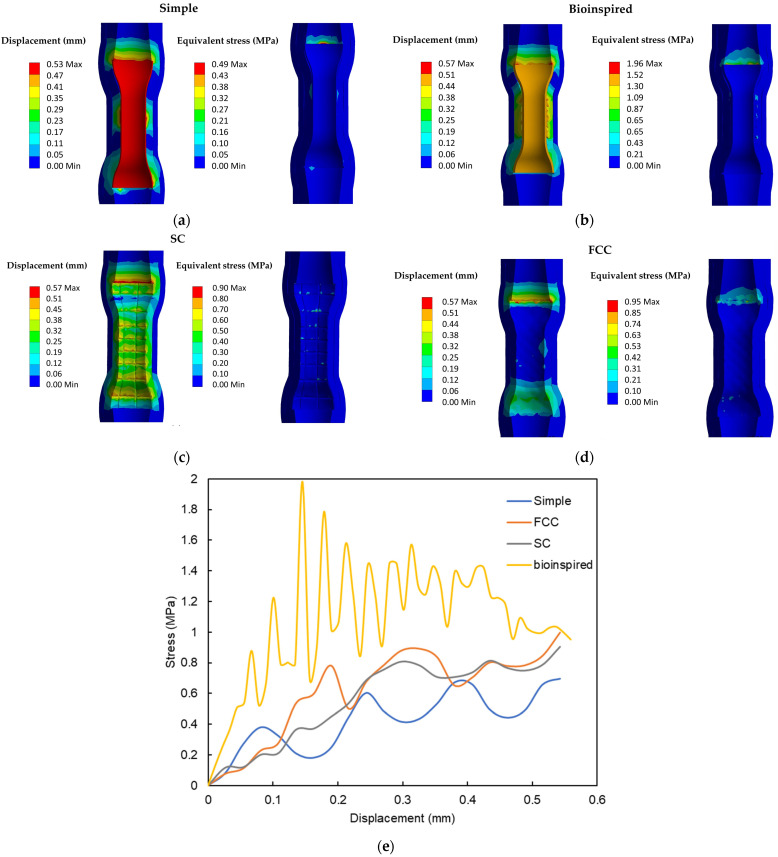
(**a**–**d**) The deformation response and distribution of stress during the final stage of the anti-migration simulation was analyzed using an FEA of the simple, bioinspired, SC, and FCC stents and (**e**) the stress–displacement curves of the novel biomimetic stent.

The anti-migration simulation test highlighted the total deformation, as shown in Figure 12a–d, and a concentration of stress primarily at the tube, as indicated by the equivalent von Mises stress results. This revealed the distribution of stress in the FEA model, simulating the stents’ behavior during the final phase of the anti-migration test. Figure 12e presents the stress–displacement responses for the various stent configurations. The maximum stress experienced during the process of slippage was calculated to be 0.49 MPa for the simple configuration, 0.96 MPa for the FCC configuration, 0.91 MPa for the SC configuration, and 1.96 MPa for the novel bioinspired configuration. No concerns arose regarding the concentration of stress at the tangent area of the stents for all configurations, as the tensile yield strength was previously determined to be 20 ± 5 MPa.

## 4. Discussion and Conclusions

Many esophageal diseases are considered to be treated by effective esophageal stenting. However, SEPS and SEMS are associated with certain complications, such as migration, perforation, and food impaction, which may require removal or replacement of the stent. Over the past few years, biodegradable stents have emerged as a promising alternative to SEPS and SEMS, eliminating the need for the stent’s removal or replacement.

Three-dimensional printing has several advantages over conventional fabrication methods in manufacturing esophageal stents. One advantage is the ability to produce complex geometries with high precision, which is difficult or impossible to achieve with conventional methods. Another advantage is the ability to produce patient-specific stents, which can improve treatment outcomes and reduce the risk of complications. Additionally, 3D printing allows for faster prototyping and production, which can save time and reduce costs. Three-dimensional printing can also produce stents with unique properties, such as controlled drug release, which may improve treatment efficacy and patient outcomes.

Thermoplastic polyurethane (TPU) is a biocompatible material that does not cause any harm to the body, making it a suitable material for medical implants such as stents. It is flexible and can conform to the shape of the esophagus, allowing for a more comfortable fit for the patient. TPU offers strong mechanical qualities such á high tensile strength and flexibility, which are crucial for the lifetime and endurance of stents. It can be easily 3D printed, which allows for customized stent designs and faster fabrication times compared with traditional methods of manufacturing stents.

The compression–recovery test demonstrated that all stent designs exhibited good mechanical stability by recovering to nearly their original diameter after compression, with no permanent deformation observed. This indicated that the 3D printed TPU can withstand the constrained delivery profile without sacrificing its ability to re-expand adequately.

Evaluating the self-expansion capability revealed differences among the designs. While all stents expanded to diameters that were sufficient for esophageal scaffolding, the novel bioinspired geometry exhibited the largest middle diameter of 17.02 mm. This enhanced radial expansion could improve wall apposition and provide greater outward force to open occluded strictures. However, the conventional cylindrical stent displayed the smallest expansion, both centrally and at the ends, suggesting more limited self-expansion ability. Critically, the bioinspired design demonstrated superior anchoring ability by requiring 1.32 N of force to prevent migration, approaching the optimal ~1.5 N benchmark from Maohua Lin et al. for esophageal stenting applications. The FCC (0.88 N) and SC (0.87 N) designs also improved over the simple cylindrical stent (0.5 N) but did not match the bioinspired geometry’s anti-migration performance. Maximizing this anchoring capability reduces the risk of dislocation during prolonged implantation. Further accelerated aging studies under simulated esophageal conditions are needed to estimate the functional lifetime and to monitor the degradation of the 3D printed stent’s mechanical properties, surface integrity, and structural stability over time. Incorporating anti-microbial agents into the polymeric ink allows localized drug delivery to potentially prevent periprosthetic infections. Three-dimensional printing enables one to tailor the kinetics of release through customization of the geometry and the material’s composition. This 3D printing approach provides opportunities for personalized drug delivery devices by controlling the release rates and temporal profiles based on integrated computational modeling and patient-specific data.

In conclusion, this work demonstrated that by applying biomimetic design principles and leveraging additive manufacturing technology, we were able to optimize the geometric configuration of 3D printed esophageal stents for improved mechanical performance and conformability to the esophageal anatomy, based on in vitro evaluations. However, further in vivo studies are needed to validate the implications of the enhanced clinical performance of this bioinspired stent design. The novel bioinspired stent exhibited superior characteristics compared with conventional cylindrical geometries, including improved radial expansion forces to maximize patency and anti-migration anchoring forces, closely matching the optimal benchmark for resisting dislocation. By harnessing biomimicry, an optimized patient-specific stent design was realized with the potential to address the key challenges faced by the currently available esophageal stents. This biomimetic approach presents promising opportunities for next-generation medical devices designed for multiple clinical applications. Moreover, new insights were gained that should assist a manufacturer to optimize their potential esophageal stents. Nonetheless, further research is required. Future work includes investigation of the use of other additive manufacturing techniques, such as selective laser sintering (SLS), for fabricating esophageal stents without the need for the removal of supports; the development of new stent designs that improve their compressibility and resistance to migration; the exploration of the use of other biocompatible materials for 3D printing esophageal stents, such as poly-p-diaxanon (PDO); and conducting the corresponding experiments to evaluate their degradation process. Moreover, further research should focus on the development of computational models to simulate the deployment and performance of the esophageal stents in the esophagus and evaluate their efficacy under different conditions; investigation of the long-term effects of the esophageal stents on the esophageal tissue, such as inflammation, fibrosis, or tissue remodeling; and investigation of the in vivo performance of the 3D printed esophageal stents in animal models or human clinical trials.

## Figures and Tables

**Table 1:** The stent’s design variables.

Name	Variable	Value (mm)
Shaft’s diameter	D1	10
Flare’s diameter	D2	16
Shaft’s length	L1	25
Total length	L2	31
Fillet radius	R2	8
Thickness	T	0.4

**Table 2:** The suction cup’s design variables.

Name	Variable	Value (mm)
Top diameter	D3	0.6
Base diameter	D4	0.3
Suction cup’s length	L3	0.8
Cavity’s radius	R2	0.45

**Table 3:** The bioinspired stent’s design variables.

Name	Variable	Value
Number of rows	N	4
Center distance	H1	3 (mm)
Center angle	θ	36 (°)

**Table 4:** FFF parameters.

Parameter	Value
Nozzle	0.2 mm
Layer height	0.1 mm
Infill density	100%
Infill pattern	Concentric
Printing temperature	190 °C
Build plate temperature	50 °C
Flow	100%
Print speed	50 mm/s
Fan speed	100%
Support placement	Everywhere
Support Z distance	0.1 mm

**Table 5:** Mooney–Rivlin model with five parameters obtained by curve-fitting of the experimental results.

	$C_{10}$ (MPa)	$C_{01}$ (MPa)	$C_{20}$ (MPa)	$C_{11}$ (MPa)	$C_{02}$ (MPa)
Muscle	0.0174	−0.01874	0.00129	0.01101	−0.01323
Mucosa–submucosa	−0.07542	0.08198	0.11805	−0.30022	0.24042
TPU	−0.122	0.035	1.79	−0.112	−0.0538

## Data Availability

The data that support the findings of this study are available from the corresponding authors upon request.
